# Certain new bounds considering the weighted Simpson-like type inequality and applications

**DOI:** 10.1186/s13660-018-1924-3

**Published:** 2018-12-04

**Authors:** Chun-Yan Luo, Ting-Song Du, Mehmet Kunt, Yao Zhang

**Affiliations:** 1 Department of Mathematics, College of Science, China Three Gorges University, Yichang, China; 2 Three Gorges Mathematical Research Center, China Three Gorges University, Yichang, China; 3 Department of Mathematics, Faculty of Sciences, Karadeniz Technical University, Trabzon, Turkey

**Keywords:** 26A33, 41A55, 26D15, 26E60, Simpson’s inequality, $(\alpha,m,h)$-convex function, $(\alpha,m,s)$-convex function

## Abstract

We investigate a weighted Simpson-type identity and obtain new estimation-type results related to the weighted Simpson-like type inequality for the first-order differentiable mappings. We also present some applications to *f*-divergence measures and to higher moments of continuous random variables.

## Introduction and preliminaries

The following inequality is named the Simpson integral inequality:

1.1 $$ \begin{aligned}[b] & \biggl\vert \frac{1}{6} \biggl[f(r_{1})+4f \biggl(\frac{r_{1}+r_{2}}{2} \biggr)+f(r_{2}) \biggr]-\frac{1}{r_{2}-r_{1}} \int^{r_{2}}_{r_{1}} f(x)\,\mathrm{d}x \biggr\vert \\ &\quad \leq\frac{1}{2880} \bigl\Vert f^{(4)} \bigr\Vert _{\infty}(r_{2}-r_{1})^{4}, \end{aligned} $$

where $f:[r_{1},r_{2}]\rightarrow\mathbb{R}$ is a four times continuously differentiable mapping on $(r_{1},r_{2})$, and $\| f^{(4)} \|_{\infty}=\mathrm{sup}_{t\in(r_{1},r_{2})} |f^{(4)}(t) |<\infty$.

To see more recent results and the related generalizations with respect to (1.1), we refer the readers to [1–3, 5–18, 22–26, 29–34] and the references therein.

Let us recall that Miheşan [20] presented a class of mappings, called $(\alpha,m)$-convex functions, as follows: A mapping $f:[0,b^{*}] \rightarrow\mathbb{R}$, $b^{*}>0$, is said to be $(\alpha ,m)$-convex if

$$ f \bigl(\lambda x+m(1-\lambda)y \bigr)\leq\lambda^{\alpha} f(x)+m\bigl(1- \lambda^{\alpha}\bigr)f(y) $$

for all $x,y\in[0,b^{*}]$ and $\lambda\in[0,1]$ with some fixed $(\alpha,m)\in(0,1]\times(0,1]$. Shuang et al. [28] proved the following result for such mappings.

### Theorem 1.1

*Let* $f:\mathbb{R}_{0}=[0,\infty)\rightarrow\mathbb{R}$ *be a differentiable function on* $\mathbb{R}_{0}$, *let* $r_{1},r_{2}\in\mathbb {R}_{0}$, $r_{1}< r_{2}$, *and let* $f'\in L^{1}[r_{1},r_{2}]$. *If* $|f'|^{q}$ *is* $(\alpha ,m)$-*convex on* $[0,\frac{r_{2}}{m}]$ *for* $(\alpha,m)\in(0,1]\times(0,1]$ *and* $q>1$, *then*

$$ \begin{aligned} & \biggl\vert \frac{1}{8} \biggl[f(r_{1})+6f \biggl(\frac{r_{1}+r_{2}}{2} \biggr)+f(r_{2}) \biggr]-\frac{1}{r_{2}-r_{1}} \int_{r_{1}}^{r_{2}}f(x)\,\mathrm{d}x \biggr\vert \\ &\quad \leq\frac{r_{2}-r_{1}}{4} \biggl(\frac{(q-1) (3^{(2q-1)/(q-1)}+1 )}{(2q-1)2^{2(2q-1)/(q-1)}} \biggr)^{1-\frac{1}{q}} \\ &\qquad {} \times \biggl\{ \biggl[\frac{1}{1+\alpha} \bigl\vert f'(r_{1}) \bigr\vert ^{q}+ \biggl( \frac {m\alpha}{1+\alpha} \biggr) \biggl\vert f' \biggl( \frac{r_{1}+r_{2}}{2m} \biggr) \biggr\vert ^{q} \biggr]^{\frac{1}{q}} \\ & \qquad {}+ \biggl[\frac{1}{1+\alpha} \biggl\vert f' \biggl( \frac{r_{1}+r_{2}}{2} \biggr) \biggr\vert ^{q}+ \biggl(\frac{m\alpha}{1+\alpha} \biggr) \biggl\vert f' \biggl(\frac{r_{2}}{m} \biggr) \biggr\vert ^{q} \biggr]^{\frac{1}{q}} \biggr\} . \end{aligned} $$

Noor et al. [21], introduced the class of $(\alpha ,m,h)$-convex functions that unifies several new and known classes of convex functions as follows.

### Definition 1.1

([21])

Let $h: J\subseteq\mathbb{R}\rightarrow\mathbb{R}$. A function $f:I\subseteq\mathbb{R}\rightarrow(0,\infty)$ is said to be $(\alpha ,m,h)$-convex function if

1.2 $$ \begin{aligned} f \bigl(tx+m(1-t)y \bigr)\leq h \bigl(t^{\alpha}\bigr) f(x)+mh\bigl(1-t^{\alpha}\bigr)f(y) \end{aligned} $$

for all $x,y\in I$ and $t\in[0,1]$ with some fixed $(\alpha,m)\in (0,1]\times(0,1]$.

Note that in [21] the authors have forgotten to write the second *m* in (1.2) in their original definition.

Let us discuss several particular cases of Definition 1.1.

I. If $h(t)=t^{s}$ for $s\in (0,1]$, then Definition 1.1 reduces to the definition of $(\alpha,m,s)$-convexity.
II. If $h(t)=t^{s}$ for $s\in (0,1]$ and $\alpha=1$, then Definition 1.1 reduces to the definition of $(s,m)$-convexity.
III. If $h(t)=t$, then Definition 1.1 reduces to the definition of $(\alpha,m)$-convexity.
IV. If $h(t)=1$, then Definition 1.1 reduces to the definition of $(m,P)$-convexity.
V. If $h(t)=t(1-t)$ and $\alpha =1$, then Definition 1.1 reduces to the definition of $(m, tgs)$-convexity.
VI. If $h(t)=\frac{\sqrt {1-t}}{2\sqrt{t}}$ and $\alpha=1$, then Definition 1.1 reduces to the definition of *m*-*MT*-convexity.

Also, the following theorem was proved in [19]. It obtains an estimation-type result associated with the weighted Simpson-type inequality for *h*-convex mappings using Hölder’s inequality.

### Theorem 1.2

*Let* $f: [r_{1},r_{2}]\rightarrow\mathbb{R}$ *be a differentiable function on* $(r_{1},r_{2})$ *such that* $f'\in L^{1}[r_{1},r_{2}]$, *and let* $w: [r_{1},r_{2}]\rightarrow\mathbb{R}$ *be continuous and symmetric with respect to* $\frac{r_{1}+r_{2}}{2}$. *If* $|f'|^{q}$ *is* *h*-*convex on* $[r_{1},r_{2}]$ *for* $q>1$ *and* $p^{-1}+q^{-1}=1$, *then*

$$ \begin{aligned} & \biggl\vert \frac{1}{6(r_{2}-r_{1})} \biggl[f(r_{1})+4f \biggl(\frac{r_{1}+r_{2}}{2} \biggr)+f(r_{2}) \biggr] \int_{r_{1}}^{r_{2}}w(x)\,\mathrm{d}x-\frac{1}{r_{2}-r_{1}} \int _{r_{1}}^{r_{2}}w(x)f(x)\,\mathrm{d}x \biggr\vert \\ &\quad \leq\frac{r_{2}-r_{1}}{12} \Vert w \Vert _{[r_{1},r_{2}],\infty}\cdot \biggl( \frac {1+2^{p+1}}{3(p+1)} \biggr)^{\frac{1}{p}}\cdot2^{\frac{1}{q}} \\ & \qquad {}\times \biggl\{ \biggl[ \bigl\vert f'(r_{1}) \bigr\vert ^{q} \int_{0}^{\frac {1}{2}}h(t)\,\mathrm{d}t+ \bigl\vert f'(r_{2}) \bigr\vert ^{q} \int_{\frac {1}{2}}^{1}h(t)\,\mathrm{d}t \biggr]^{\frac{1}{q}} \\ & \qquad {}+ \biggl[ \bigl\vert f'(r_{1}) \bigr\vert ^{q} \int_{\frac{1}{2}}^{1}h(t)\,\mathrm{d}t+ \bigl\vert f'(r_{2}) \bigr\vert ^{q} \int_{0}^{\frac{1}{2}}h(t)\,\mathrm{d}t \biggr]^{\frac{1}{q}} \biggr\} . \end{aligned} $$

Different from [19] and [28], our purpose in this paper is to give some new bounds related to the weighted Simpson-like type inequality for the first-order differentiable mappings.

To obtain the principal results, we presume that the absolute value of the derivative of the considered mapping is $(\alpha,m,h)$-convex. Next, we substitute this hypothesis with the boundedness of the derivative and with a Lipschitz condition for the derivative of the considered mapping to establish integral inequalities with new estimation-type results. Also, we provide some applications to *f*-divergence measures and to higher moments of continuous random variables.

## Main results

To obtain our main results, we need the following lemma.

### Lemma 2.1

*Let* $f: I\subseteq\mathbb{R}\rightarrow\mathbb{R}$ *be a differentiable function on* $I^{\circ}$, $a,b\in I^{\circ}$ *with* $a< b$, *and let* $w: [a,b]\rightarrow\mathbb{R}$ *be symmetric with respect to* $\frac{a+b}{2}$. *If* $f',w\in L^{1}[a,b]$, *then*

2.1 $$ \begin{aligned}[b] &\frac{1}{8(b-a)} \biggl[f(a)+6f \biggl(\frac{a+b}{2} \biggr)+f(b) \biggr] \int _{a}^{b}w(x)\,\mathrm{d}x-\frac{1}{b-a} \int_{a}^{b}w(x)f(x)\,\mathrm{d}x \\ &\quad =\frac{b-a}{4} \biggl\{ \int_{0}^{1}p_{1}(t)f' \biggl(ta+(1-t)\frac {a+b}{2} \biggr)\,\mathrm{d}t \\ &\qquad {}+ \int_{0}^{1}p_{2}(t)f' \biggl(t\frac {a+b}{2}+(1-t)b \biggr)\,\mathrm{d}t \biggr\} ,\end{aligned} $$

*where*

$$ \begin{aligned} p_{1}(t)=\frac{3}{4} \int_{0}^{1}w \biggl(sa+(1-s)\frac{a+b}{2} \biggr)\,\mathrm{d}s- \int_{0}^{t}w \biggl(sa+(1-s)\frac{a+b}{2} \biggr)\,\mathrm{d}s \end{aligned} $$

*and*

$$ \begin{aligned} p_{2}(t)=\frac{1}{4} \int_{0}^{1}w \biggl(s\frac{a+b}{2}+(1-s)b \biggr)\,\mathrm{d}s- \int_{0}^{t}w \biggl(s\frac{a+b}{2}+(1-s)b \biggr)\,\mathrm{d}s. \end{aligned} $$

### Proof

Integrating by parts and changing the variables, we have

$$ \begin{aligned} \mathcal{I}_{1}={}& \int_{0}^{1}p_{1}(t)f' \biggl(ta+(1-t)\frac{a+b}{2} \biggr)\,\mathrm{d}t \\ ={}& \int_{0}^{1} \biggl[\frac{3}{4} \int_{0}^{1}w \biggl(sa+(1-s)\frac {a+b}{2} \biggr)\,\mathrm{d}s \\ & - \int_{0}^{t}w \biggl(sa+(1-s)\frac{a+b}{2} \biggr)\,\mathrm{d}s \biggr]f' \biggl(ta+(1-t)\frac{a+b}{2} \biggr)\,\mathrm{d}t \\ ={}&\frac{-2}{b-a} \biggl[\frac{3}{4} \int_{0}^{1}w \biggl(sa+(1-s)\frac {a+b}{2} \biggr)\,\mathrm{d}s \\ &{} - \int_{0}^{t}w \biggl(sa+(1-s)\frac{a+b}{2} \biggr)\,\mathrm{d}s \biggr]f \biggl(ta+(1-t)\frac{a+b}{2} \biggr)\bigg|_{0}^{1} \\ & {}-\frac{2}{b-a} \int_{0}^{1}w \biggl(ta+(1-t)\frac{a+b}{2} \biggr)f \biggl(ta+(1-t)\frac{a+b}{2} \biggr)\,\mathrm{d}t \\ ={}&\frac{-2}{b-a} \biggl[-\frac{1}{4}f(a)-\frac{3}{4}f \biggl(\frac {a+b}{2} \biggr) \biggr] \int_{0}^{1}w \biggl(sa+(1-s)\frac{a+b}{2} \biggr)\,\mathrm{d}s \\ &{} -\frac{2}{b-a} \int_{0}^{1}w \biggl(ta+(1-t)\frac{a+b}{2} \biggr)f \biggl(ta+(1-t)\frac{a+b}{2} \biggr)\,\mathrm{d}t \\ ={}&\frac{1}{(b-a)^{2}} \biggl[f(a)+3f \biggl(\frac{a+b}{2} \biggr) \biggr] \int _{a}^{\frac{a+b}{2}}w(x)\,\mathrm{d}x -\frac{4}{(b-a)^{2}} \int_{a}^{\frac{a+b}{2}}w(x)f(x)\,\mathrm{d}x. \end{aligned} $$

Similarly, we get

$$ \begin{aligned} \mathcal{I}_{2}={}& \int_{0}^{1}p_{2}(t)f' \biggl(t\frac{a+b}{2}+(1-t)b \biggr)\,\mathrm{d}t \\ ={}&\frac{-2}{b-a} \biggl[\frac{1}{4} \int_{0}^{1}w \biggl(s\frac {a+b}{2}+(1-s)b \biggr)\,\mathrm{d}s \\ &{} - \int_{0}^{t}w \biggl(s\frac{a+b}{2}+(1-s)b \biggr)\,\mathrm{d}s \biggr]f \biggl(t\frac{a+b}{2}+(1-t)b \biggr)\bigg|_{0}^{1} \\ &{} -\frac{2}{b-a} \int_{0}^{1}w \biggl(t\frac{a+b}{2}+(1-t)b \biggr)f \biggl(t\frac {a+b}{2}+(1-t)b \biggr)\,\mathrm{d}t \\ ={}&\frac{1}{(b-a)^{2}} \biggl[3f \biggl(\frac{a+b}{2} \biggr)+f(b) \biggr] \int_{\frac {a+b}{2}}^{b}w(x)\,\mathrm{d}x-\frac{4}{(b-a)^{2}} \int_{\frac {a+b}{2}}^{b}w(x)f(x)\,\mathrm{d}x. \end{aligned} $$

Since $w(x)$ is symmetric with respect to $\frac{a+b}{2}$, we have

$$\int_{a}^{\frac{a+b}{2}}w(x)\,\mathrm{d}x= \int_{\frac {a+b}{2}}^{b}w(x)\,\mathrm{d}x=\frac{1}{2} \int_{a}^{b}w(x)\,\mathrm{d}x. $$

Thus we have

$$ \begin{aligned} &\frac{b-a}{4} (\mathcal{I}_{1}+ \mathcal{I}_{2} ) \\ &\quad =\frac{1}{8(b-a)} \biggl[f(a)+6f \biggl(\frac{a+b}{2} \biggr)+f(b) \biggr] \int _{a}^{b}w(x)\,\mathrm{d}x-\frac{1}{b-a} \int_{a}^{b}w(x)f(x)\,\mathrm{d}x, \end{aligned} $$

which completes the proof. □

Throughout the work, we write $\|w\|_{[a,b],\infty}=\sup_{x\in [a,b]}|w(x)|$ for a continuous mapping $w:[a,b]\rightarrow\mathbb{R}$. Next, we derive our main results.

### Theorem 2.1

*Let* $f: \mathbb{R}_{0}=[0,\infty)\rightarrow\mathbb{R}$ *be a differentiable function on* $\mathbb{R}_{0}$, $a,b\in \mathbb{R}_{0}$, $a< b$, *let* $f'\in L^{1}[a,b]$, *and let* $w: [a,b]\rightarrow\mathbb{R}$ *be continuous and symmetric with respect to* $\frac{a+b}{2}$. *If* $|f'|^{q}$ *for* $q\geq1$ *is* $(\alpha,m,h)$-*convex on* $[0,\frac{b}{m}]$ *with some fixed* $(\alpha,m)\in(0,1]\times(0,1]$, *then*

2.2 $$ \begin{aligned}[b] & \biggl\vert \frac{1}{8(b-a)} \biggl[f(a)+6f \biggl(\frac{a+b}{2} \biggr)+f(b) \biggr] \int _{a}^{b}w(x)\,\mathrm{d}x-\frac{1}{b-a} \int_{a}^{b}w(x)f(x)\,\mathrm{d}x \biggr\vert \\ &\quad \leq\frac{b-a}{4} \Vert w \Vert _{[a,b],\infty} \biggl( \frac{5}{16} \biggr)^{1-\frac {1}{q}} \\ &\qquad {} \times \biggl\{ \biggl[ \int_{0}^{1} \biggl\vert \frac{3}{4}-t \biggr\vert \biggl(h\bigl(t^{\alpha}\bigr) \bigl\vert f'(a) \bigr\vert ^{q}+h\bigl(1-t^{\alpha}\bigr)m \biggl\vert f' \biggl(\frac {a+b}{2m} \biggr) \biggr\vert ^{q} \biggr)\,\mathrm{d}t \biggr]^{\frac{1}{q}} \\ &\qquad {} + \biggl[ \int_{0}^{1} \biggl\vert \frac{1}{4}-t \biggr\vert \biggl(h\bigl(t^{\alpha}\bigr) \biggl\vert f' \biggl(\frac{a+b}{2} \biggr) \biggr\vert ^{q}+h \bigl(1-t^{\alpha}\bigr)m \biggl\vert f' \biggl( \frac {b}{m} \biggr) \biggr\vert ^{q} \biggr)\,\mathrm{d}t \biggr]^{\frac{1}{q}} \biggr\} . \end{aligned} $$

### Proof

Applying Lemma 2.1 and using the fact that $\|w\|_{[a, \frac {a+b}{2}],\infty}, \|w\|_{[\frac{a+b}{2}, b],\infty}\leq\|w\|_{[a, b],\infty}$, we have

2.3 $$ \begin{aligned}[b] & \biggl\vert \frac{1}{8(b-a)} \biggl[f(a)+6f \biggl(\frac{a+b}{2} \biggr)+f(b) \biggr] \int_{a}^{b}w(x)\,\mathrm{d}x-\frac{1}{b-a} \int_{a}^{b}w(x)f(x)\,\mathrm{d}x \biggr\vert \\ &\quad \leq\frac{b-a}{4} \biggl\{ \int_{0}^{1} \biggl\vert \frac{3}{4} \int_{0}^{1}w \biggl(sa+(1-s)\frac{a+b}{2} \biggr)\,\mathrm{d}s \\ &\qquad {} - \int_{0}^{t}w \biggl(sa+(1-s)\frac{a+b}{2} \biggr)\,\mathrm{d}s \biggr\vert \biggl\vert f' \biggl(ta+(1-t) \frac{a+b}{2} \biggr) \biggr\vert \,\mathrm{d}t \\ &\qquad {} + \int_{0}^{1} \biggl\vert \frac{1}{4} \int_{0}^{1}w \biggl(s\frac {a+b}{2}+(1-s)b \biggr)\,\mathrm{d}s \\ & \qquad {}- \int_{0}^{t}w \biggl(s\frac{a+b}{2}+(1-s)b \biggr)\,\mathrm{d}s \biggr\vert \biggl\vert f' \biggl(t \frac{a+b}{2}+(1-t)b \biggr) \biggr\vert \,\mathrm{d}t \biggr\} \\ &\quad \leq\frac{b-a}{4} \Vert w \Vert _{[a,b],\infty} \biggl\{ \int_{0}^{1} \biggl\vert \frac {3}{4} \int_{0}^{1}\,\mathrm{d}s- \int_{0}^{t}\,\mathrm{d}s \biggr\vert \biggl\vert f' \biggl(ta+(1-t)\frac{a+b}{2} \biggr) \biggr\vert \,\mathrm{d}t \\ & \qquad {}+ \int_{0}^{1} \biggl\vert \frac{1}{4} \int_{0}^{1}\,\mathrm{d}s- \int _{0}^{t}\,\mathrm{d}s \biggr\vert \biggl\vert f' \biggl(t\frac{a+b}{2}+(1-t)b \biggr) \biggr\vert \,\mathrm{d}t \biggr\} \\ &\quad =\frac{b-a}{4} \Vert w \Vert _{[a,b],\infty} \biggl\{ \int_{0}^{1} \biggl\vert \frac {3}{4}-t \biggr\vert \biggl\vert f' \biggl(ta+(1-t)\frac{a+b}{2} \biggr) \biggr\vert \,\mathrm{d}t \\ &\qquad {} + \int_{0}^{1} \biggl\vert \frac{1}{4}-t \biggr\vert \biggl\vert f' \biggl(t\frac {a+b}{2}+(1-t)b \biggr) \biggr\vert \,\mathrm{d}t \biggr\} . \end{aligned} $$

Using the power mean inequality, we have

2.4 $$\begin{aligned}& \biggl\vert \frac{1}{8(b-a)} \biggl[f(a)+6f \biggl(\frac{a+b}{2} \biggr)+f(b) \biggr] \int_{a}^{b}w(x)\,\mathrm{d}x-\frac{1}{b-a} \int_{a}^{b}w(x)f(x)\,\mathrm{d}x \biggr\vert \\& \quad \leq\frac{b-a}{4} \Vert w \Vert _{[a,b],\infty} \\& \qquad {} \times \biggl\{ \biggl( \int_{0}^{1} \biggl\vert \frac{3}{4}-t \biggr\vert \,\mathrm{d}t \biggr)^{1-\frac{1}{q}} \biggl[ \int_{0}^{1} \biggl\vert \frac{3}{4}-t \biggr\vert \biggl\vert f' \biggl(ta+(1-t)\frac{a+b}{2} \biggr) \biggr\vert ^{q}\,\mathrm{d}t \biggr]^{\frac{1}{q}} \\& \qquad {} + \biggl( \int_{0}^{1} \biggl\vert \frac{1}{4}-t \biggr\vert \,\mathrm{d}t \biggr)^{1-\frac {1}{q}} \biggl[ \int_{0}^{1} \biggl\vert \frac{1}{4}-t \biggr\vert \biggl\vert f' \biggl(t\frac {a+b}{2}+(1-t)b \biggr) \biggr\vert ^{q}\,\mathrm{d}t \biggr]^{\frac{1}{q}} \biggr\} . \end{aligned}$$

From (2.3) and (2.4) we get the inquired inequality in (2.2), since

2.5 $$ \begin{aligned} & \int_{0}^{1} \biggl\vert \frac{1}{4}-t \biggr\vert \,\mathrm{d}t = \int_{0}^{1} \biggl\vert \frac{3}{4}-t \biggr\vert \,\mathrm{d}t =\frac{5}{16}, \end{aligned} $$

and using the $(\alpha,m,h)$-convexity of $|f'|^{q}$ on $[0,\frac {b}{m}]$, we have

2.6 $$ \begin{aligned} \biggl\vert f' \biggl(ta+(1-t)\frac{a+b}{2} \biggr) \biggr\vert ^{q}\leq h \bigl(t^{\alpha}\bigr) \bigl\vert f'(a) \bigr\vert ^{q}+h\bigl(1-t^{\alpha}\bigr)m \biggl\vert f' \biggl(\frac{a+b}{2m} \biggr) \biggr\vert ^{q} \end{aligned} $$

and

2.7 $$ \begin{aligned} \biggl\vert f' \biggl(t \frac{a+b}{2}+(1-t)b \biggr) \biggr\vert ^{q}\leq h \bigl(t^{\alpha}\bigr) \biggl\vert f' \biggl( \frac{a+b}{2} \biggr) \biggr\vert ^{q}+h\bigl(1-t^{\alpha}\bigr)m \biggl\vert f' \biggl(\frac {b}{m} \biggr) \biggr\vert ^{q}. \end{aligned} $$

Direct computation provides the following cases. □

### Corollary 2.1

*If we take* $q=1$ *in Theorem* 2.1, *then we have the following inequality for* $(\alpha,m,h)$-*convex functions*:

$$ \begin{aligned} & \biggl\vert \frac{1}{8(b-a)} \biggl[f(a)+6f \biggl(\frac{a+b}{2} \biggr)+f(b) \biggr] \int _{a}^{b}w(x)\,\mathrm{d}x-\frac{1}{b-a} \int_{a}^{b}w(x)f(x)\,\mathrm{d}x \biggr\vert \\ &\quad \leq\frac{b-a}{4} \Vert w \Vert _{[a,b],\infty} \biggl\{ \int_{0}^{1} \biggl\vert \frac {3}{4}-t \biggr\vert \biggl(h\bigl(t^{\alpha}\bigr) \bigl\vert f'(a) \bigr\vert +h\bigl(1-t^{\alpha}\bigr)m \biggl\vert f' \biggl(\frac{a+b}{2m} \biggr) \biggr\vert \biggr)\, \mathrm{d}t \\ &\qquad {} + \int_{0}^{1} \biggl\vert \frac{1}{4}-t \biggr\vert \biggl(h\bigl(t^{\alpha}\bigr) \biggl\vert f' \biggl(\frac{a+b}{2} \biggr) \biggr\vert +h\bigl(1-t^{\alpha}\bigr)m \biggl\vert f' \biggl(\frac{b}{m} \biggr) \biggr\vert \biggr)\,\mathrm{d}t \biggr\} . \end{aligned} $$

### Remark 2.1

Consider Corollary 2.1.

(i) Putting $h(t)=1$, we have the following inequality for $(m,P)$-convex functions:
  $$ \begin{aligned} & \biggl\vert \frac{1}{8(b-a)} \biggl[f(a)+6f \biggl(\frac{a+b}{2} \biggr)+f(b) \biggr] \int _{a}^{b}w(x)\,\mathrm{d}x-\frac{1}{b-a} \int_{a}^{b}w(x)f(x)\,\mathrm{d}x \biggr\vert \\ &\quad \leq\frac{5(b-a)}{64} \Vert w \Vert _{[a,b],\infty} \\ &\qquad {}\times \biggl\{ \biggl[ \bigl\vert f'(a) \bigr\vert + \biggl\vert f' \biggl(\frac{a+b}{2} \biggr) \biggr\vert \biggr] +m \biggl[ \biggl\vert f' \biggl(\frac{a+b}{2m} \biggr) \biggr\vert + \biggl\vert f' \biggl(\frac{b}{m} \biggr) \biggr\vert \biggr] \biggr\} . \end{aligned} $$
(ii) Putting $h(t)=t(1-t)$ and $\alpha=1$, we have the following inequality for $(m, tgs)$-convex functions:
  $$ \begin{aligned} & \biggl\vert \frac{1}{8(b-a)} \biggl[f(a)+6f \biggl(\frac{a+b}{2} \biggr)+f(b) \biggr] \int _{a}^{b}w(x)\,\mathrm{d}x-\frac{1}{b-a} \int_{a}^{b}w(x)f(x)\,\mathrm{d}x \biggr\vert \\ &\quad \leq\frac{71(b-a)}{3\cdot2^{11}} \Vert w \Vert _{[a,b],\infty} \biggl\{ \biggl[ \bigl\vert f'(a) \bigr\vert + \biggl\vert f' \biggl(\frac{a+b}{2} \biggr) \biggr\vert \biggr] \\ &\qquad {}+m \biggl[ \biggl\vert f' \biggl(\frac{a+b}{2m} \biggr) \biggr\vert + \biggl\vert f' \biggl(\frac{b}{m} \biggr) \biggr\vert \biggr] \biggr\} . \end{aligned} $$
(iii) Putting $h(t)=t^{s}$ and using the inequality $(1-t^{\alpha})^{s}\leq 2^{1-s}-t^{s\alpha}$ for $t\in[0,1]$ with some fixed $\alpha\in(0,1]$, $s\in(0,1]$, we have the following inequality for $(\alpha,m,s)$-convex functions:
  $$ \begin{aligned} & \biggl\vert \frac{1}{8(b-a)} \biggl[f(a)+6f \biggl(\frac{a+b}{2} \biggr)+f(b) \biggr] \int _{a}^{b}w(x)\,\mathrm{d}x-\frac{1}{b-a} \int_{a}^{b}w(x)f(x)\,\mathrm{d}x \biggr\vert \\ &\quad \leq\frac{b-a}{4} \Vert w \Vert _{[a,b],\infty} \biggl\{ \biggl[ \Delta_{1} \bigl\vert f'(a) \bigr\vert + \biggl( \frac{5\cdot2^{1-s}}{16}-\Delta_{1} \biggr)m \biggl\vert f' \biggl(\frac{a+b}{2m} \biggr) \biggr\vert \biggr] \\ & \qquad {}+ \biggl[\Delta_{2} \biggl\vert f' \biggl( \frac{a+b}{2} \biggr) \biggr\vert + \biggl(\frac{5\cdot 2^{1-s}}{16}- \Delta_{2} \biggr)m \biggl\vert f' \biggl( \frac{b}{m} \biggr) \biggr\vert \biggr] \biggr\} , \end{aligned} $$

where

$$ \begin{aligned} &\Delta_{1}=\frac{3^{s\alpha+2}+2^{2s\alpha+1}s\alpha-2^{2s\alpha +2}}{2^{2s\alpha+3}(s\alpha+1)(s\alpha+2)}, \\ & \Delta_{2}=\frac{1+2^{2\alpha s+1}(3\alpha s+2)}{2^{2\alpha s+3}(s\alpha +1)(s\alpha+2)}. \end{aligned} $$

### Theorem 2.2

*Suppose that all assumptions of Theorem* 2.1 *are satisfied*. *Then*

2.8 $$ \begin{aligned}[b] & \biggl\vert \frac{1}{8(b-a)} \biggl[f(a)+6f \biggl(\frac{a+b}{2} \biggr)+f(b) \biggr] \int_{a}^{b}w(x)\,\mathrm{d}x-\frac{1}{b-a} \int_{a}^{b}w(x)f(x)\,\mathrm{d}x \biggr\vert \\ &\quad \leq\frac{b-a}{4} \Vert w \Vert _{[a,b],\infty} \biggl( \frac{5}{16} \biggr)^{1-\frac {1}{q}} \\ &\qquad {} \times \biggl\{ \biggl[ \int_{0}^{1} \biggl(h \biggl(1- \biggl( \frac{1-t}{2} \biggr)^{\alpha}\biggr)m \biggl\vert f' \biggl(\frac{a}{m} \biggr) \biggr\vert ^{q}+h \biggl( \biggl( \frac {1-t}{2} \biggr)^{\alpha}\biggr) \bigl\vert f'(b) \bigr\vert ^{q} \biggr)\,\mathrm{d}t \biggr]^{\frac{1}{q}} \\ & \qquad {}+ \biggl[ \int_{0}^{1} \biggl(h \biggl( \biggl( \frac{t}{2} \biggr)^{\alpha}\biggr) \bigl\vert f'(a) \bigr\vert ^{q}+h \biggl(1- \biggl(\frac{t}{2} \biggr)^{\alpha}\biggr)m \biggl\vert f' \biggl( \frac{b}{m} \biggr) \biggr\vert ^{q} \biggr)\,\mathrm{d}t \biggr]^{\frac{1}{q}} \biggr\} . \end{aligned} $$

### Proof

Noting that $ta+(1-t)\frac{a+b}{2}=\frac{1+t}{2}a+\frac{1-t}{2}b$ and using the $(\alpha,m,h)$-convexity of $|f'|^{q}$ on $[0,\frac{b}{m}]$, for any $t\in[0,1]$, we have the inequality

2.9 $$ \begin{aligned}[b] &\biggl\vert f' \biggl(ta+(1-t)\frac{a+b}{2} \biggr) \biggr\vert ^{q} \\ &\quad \leq h \biggl(1- \biggl(\frac {1-t}{2} \biggr)^{\alpha}\biggr)m \biggl\vert f' \biggl(\frac{a}{m} \biggr) \biggr\vert ^{q}+h \biggl( \biggl(\frac{1-t}{2} \biggr)^{\alpha}\biggr) \bigl\vert f'(b) \bigr\vert ^{q} \end{aligned} $$

and, similarly,

2.10 $$ \begin{aligned} \biggl\vert f' \biggl(t \frac{a+b}{2}+(1-t)b \biggr) \biggr\vert ^{q}\leq h \biggl( \biggl(\frac {t}{2} \biggr)^{\alpha}\biggr) \bigl\vert f'(a) \bigr\vert ^{q}+h \biggl(1- \biggl( \frac{t}{2} \biggr)^{\alpha}\biggr)m \biggl\vert f' \biggl(\frac{b}{m} \biggr) \biggr\vert ^{q}. \end{aligned} $$

Continuing from inequality (2.4) in the proof of Theorem 2.1 and using (2.9) and (2.10) with (2.5), we obtain the desired result in (2.8). This completes the proof. □

### Corollary 2.2

*If we take* $q=1$ *in Theorem* 2.2, *then the following inequality for* $(\alpha,m,h)$-*convex functions holds*:

$$ \begin{aligned} & \biggl\vert \frac{1}{8(b-a)} \biggl[f(a)+6f \biggl(\frac{a+b}{2} \biggr)+f(b) \biggr] \int_{a}^{b}w(x)\,\mathrm{d}x-\frac{1}{b-a} \int_{a}^{b}w(x)f(x)\,\mathrm{d}x \biggr\vert \\ &\quad \leq\frac{b-a}{4} \Vert w \Vert _{[a,b],\infty} \biggl\{ \int_{0}^{1} \biggl[h \biggl(1- \biggl( \frac{1-t}{2} \biggr)^{\alpha}\biggr)m \biggl\vert f' \biggl(\frac{a}{m} \biggr) \biggr\vert +h \biggl( \biggl( \frac{1-t}{2} \biggr)^{\alpha}\biggr) \bigl\vert f'(b) \bigr\vert \biggr]\,\mathrm{d}t \\ &\qquad {} + \int_{0}^{1} \biggl[h \biggl( \biggl( \frac{t}{2} \biggr)^{\alpha}\biggr) \bigl\vert f'(a) \bigr\vert +h \biggl(1- \biggl(\frac{t}{2} \biggr)^{\alpha}\biggr)m \biggl\vert f' \biggl(\frac {b}{m} \biggr) \biggr\vert \biggr]\,\mathrm{d}t \biggr\} . \end{aligned} $$

### Remark 2.2

Consider Corollary 2.2.

(i) Putting $h(t)=t^{s}$ for $s\in(0,1]$ and using the inequality $(1-t^{\alpha})^{s}\leq2^{1-s}-t^{s\alpha}$ for $t\in[0,1]$ with some fixed $\alpha\in(0,1]$ and $s\in(0,1]$ again, we have the following inequality for $(\alpha,m,s)$-convex functions:
  $$ \begin{aligned} & \biggl\vert \frac{1}{8(b-a)} \biggl[f(a)+6f \biggl(\frac{a+b}{2} \biggr)+f(b) \biggr] \int_{a}^{b}w(x)\,\mathrm{d}x-\frac{1}{b-a} \int_{a}^{b}w(x)f(x)\,\mathrm{d}x \biggr\vert \\ &\quad \leq\frac{b-a}{4} \Vert w \Vert _{[a,b],\infty} \biggl\{ \biggl(2^{1-s}- \biggl(\frac {2^{-s\alpha}}{1+\alpha s} \biggr) \biggr)m \biggl[ \biggl\vert f' \biggl(\frac{a}{m} \biggr) \biggr\vert + \biggl\vert f' \biggl(\frac{b}{m} \biggr) \biggr\vert \biggr] \\ &\qquad {} + \biggl(\frac{2^{-s\alpha}}{1+\alpha s} \biggr) \bigl[ \bigl\vert f'(a) \bigr\vert + \bigl\vert f'(b) \bigr\vert \bigr] \biggr\} . \end{aligned} $$
(ii) Putting $h(t)=1$, we have the following inequality for $(m,P)$-convex functions:
  $$ \begin{aligned} & \biggl\vert \frac{1}{8(b-a)} \biggl[f(a)+6f \biggl(\frac{a+b}{2} \biggr)+f(b) \biggr] \int_{a}^{b}w(x)\,\mathrm{d}x-\frac{1}{b-a} \int_{a}^{b}w(x)f(x)\,\mathrm{d}x \biggr\vert \\ &\quad \leq\frac{b-a}{4} \Vert w \Vert _{[a,b],\infty} \biggl\{ m \biggl[ \biggl\vert f' \biggl(\frac {a}{m} \biggr) \biggr\vert + \biggl\vert f' \biggl(\frac{b}{m} \biggr) \biggr\vert \biggr]+ \bigl[ \bigl\vert f'(a) \bigr\vert + \bigl\vert f'(b) \bigr\vert \bigr] \biggr\} . \end{aligned} $$
(iii) Putting $h(t)=t(1-t)$ and $\alpha=1$, we have the following inequality for $(m, tgs)$-convex functions:
  $$ \begin{aligned} & \biggl\vert \frac{1}{8(b-a)} \biggl[f(a)+6f \biggl(\frac{a+b}{2} \biggr)+f(b) \biggr] \int_{a}^{b}w(x)\,\mathrm{d}x-\frac{1}{b-a} \int_{a}^{b}w(x)f(x)\,\mathrm{d}x \biggr\vert \\ &\quad \leq\frac{b-a}{24} \Vert w \Vert _{[a,b],\infty} \biggl\{ m \biggl[ \biggl\vert f' \biggl(\frac {a}{m} \biggr) \biggr\vert + \biggl\vert f' \biggl(\frac{b}{m} \biggr) \biggr\vert \biggr]+ \bigl[ \bigl\vert f'(a) \bigr\vert + \bigl\vert f'(b) \bigr\vert \bigr] \biggr\} . \end{aligned} $$

The next result deals with the case where $|f'|^{q}$ for $q>1$ is $(\alpha ,m,h)$-convex.

### Theorem 2.3

*Let* $f: \mathbb{R}_{0}\rightarrow\mathbb{R}$ *be a differentiable function on* $\mathbb{R}_{0}$, $a,b\in \mathbb{R}_{0}$, $a< b$, *let* $f'\in L^{1}[a,b]$, *and let* $w: [a,b]\rightarrow\mathbb{R}$ *be continuous and symmetric with respect to* $\frac{a+b}{2}$. *If* $|f'|^{q}$ *for* $q>1$ *is* $(\alpha,m,h)$-*convex on* $[0,\frac{b}{m}]$ *with some fixed* $(\alpha,m)\in(0,1]\times(0,1]$, *then*

2.11 $$\begin{aligned}& \biggl\vert \frac{1}{8(b-a)} \biggl[f(a)+6f \biggl(\frac{a+b}{2} \biggr)+f(b) \biggr] \int _{a}^{b}w(x)\,\mathrm{d}x-\frac{1}{b-a} \int_{a}^{b}w(x)f(x)\,\mathrm{d}x \biggr\vert \\& \quad \leq\frac{b-a}{4} \Vert w \Vert _{[a,b],\infty} \mathcal{Q}^{1-\frac{1}{q}} \biggl\{ \biggl[ \int_{0}^{1} \biggl(h\bigl(t^{\alpha}\bigr) \bigl\vert f'(a) \bigr\vert ^{q}+h \bigl(1-t^{\alpha}\bigr)m \biggl\vert f' \biggl( \frac{a+b}{2m} \biggr) \biggr\vert ^{q} \biggr)\,\mathrm{d}t \biggr]^{\frac{1}{q}} \\& \qquad {} + \biggl[ \int_{0}^{1} \biggl(h\bigl(t^{\alpha}\bigr) \biggl\vert f' \biggl(\frac{a+b}{2} \biggr) \biggr\vert ^{q}+h\bigl(1-t^{\alpha}\bigr)m \biggl\vert f' \biggl(\frac{b}{m} \biggr) \biggr\vert ^{q} \biggr)\, \mathrm{d}t \biggr]^{\frac{1}{q}} \biggr\} , \end{aligned}$$

*where*

$$ \begin{aligned} \mathcal{Q}=\frac{(q-1) (3^{(2q-1)/(q-1)}+1 )}{(2q-1)2^{2(2q-1)/(q-1)}}. \end{aligned} $$

### Proof

Using the Hölder inequality for (2.3), we have

2.12 $$ \begin{aligned}[b] & \int_{0}^{1} \biggl\vert \frac{3}{4}-t \biggr\vert \biggl\vert f' \biggl(ta+(1-t)\frac {a+b}{2} \biggr)\,\mathrm{d}t \biggr\vert + \int_{0}^{1} \biggl\vert \frac{1}{4}-t \biggr\vert \biggl\vert f' \biggl(t\frac {a+b}{2}+(1-t)b \biggr)\,\mathrm{d}t \biggr\vert \\ &\quad \leq \biggl\{ \biggl( \int_{0}^{1} \biggl\vert \frac{3}{4}-t \biggr\vert ^{\frac {q}{q-1}}\,\mathrm{d}t \biggr)^{1-\frac{1}{q}} \biggl[ \int_{0}^{1} \biggl\vert f' \biggl(ta+(1-t)\frac{a+b}{2} \biggr) \biggr\vert ^{q}\, \mathrm{d}t \biggr]^{\frac{1}{q}} \\ &\qquad {} + \biggl( \int_{0}^{1} \biggl\vert \frac{1}{4}-t \biggr\vert ^{\frac{q}{q-1}}\,\mathrm{d}t \biggr)^{1-\frac{1}{q}} \biggl[ \int_{0}^{1} \biggl\vert f' \biggl(t\frac {a+b}{2}+(1-t)b \biggr) \biggr\vert ^{q}\, \mathrm{d}t \biggr]^{\frac{1}{q}} \biggr\} . \end{aligned} $$

From (2.6), (2.7), and (2.12) we get the desired inequality in (2.11), since

$$ \begin{aligned} & \int_{0}^{1} \biggl\vert \frac{3}{4}-t \biggr\vert ^{\frac{q}{q-1}}\,\mathrm{d}t = \int_{0}^{1} \biggl\vert \frac{1}{4}-t \biggr\vert ^{\frac{q}{q-1}}\,\mathrm{d}t =\frac{(q-1) (3^{(2q-1)/(q-1)}+1 )}{(2q-1)2^{2(2q-1)/(q-1)}}=\mathcal{Q}. \end{aligned} $$

 □

Now, we state some particular cases of Theorem 2.3.

### Corollary 2.3

*In Theorem* 2.3, *putting* $h(t)=t^{s}$ *and using the inequality* $(1-t^{\alpha})^{s}\leq2^{1-s}-t^{s\alpha}$ *for* $t\in[0,1]$ *with some fixed* $\alpha\in(0,1]$, $s\in(0,1]$ *again*, *we have the following inequality for* $(\alpha,m,s)$-*convex functions*:

$$ \begin{aligned} & \biggl\vert \frac{1}{8(b-a)} \biggl[f(a)+6f \biggl(\frac{a+b}{2} \biggr)+f(b) \biggr] \int _{a}^{b}w(x)\,\mathrm{d}x-\frac{1}{b-a} \int_{a}^{b}w(x)f(x)\,\mathrm{d}x \biggr\vert \\ &\quad \leq\frac{b-a}{4} \Vert w \Vert _{[a,b],\infty} \mathcal{Q}^{1-\frac{1}{q}} \biggl\{ \biggl[\frac{1}{1+s\alpha} \bigl\vert f'(a) \bigr\vert ^{q}+ \biggl(2^{1-s}- \frac {1}{1+s\alpha} \biggr)m \biggl\vert f' \biggl( \frac{a+b}{2m} \biggr) \biggr\vert ^{q} \biggr]^{\frac{1}{q}} \\ &\qquad {} + \biggl[\frac{1}{1+s\alpha} \biggl\vert f' \biggl( \frac{a+b}{2} \biggr) \biggr\vert ^{q}+ \biggl(2^{1-s}- \frac{1}{1+s\alpha} \biggr)m \biggl\vert f' \biggl( \frac{b}{m} \biggr) \biggr\vert ^{q} \biggr]^{\frac{1}{q}} \biggr\} . \end{aligned} $$

### Remark 2.3

In Corollary 2.3, if $f(a)=f (\frac{a+b}{2} )= f(b)$ with $m=1=\alpha$, then the following inequality for *s*-convex functions holds:

$$ \begin{aligned} & \biggl\vert f \biggl(\frac{a+b}{2} \biggr) \int_{a}^{b}w(x)\,\mathrm{d}x- \int _{a}^{b}w(x)f(x)\,\mathrm{d}x \biggr\vert \\ &\quad \leq\frac{(b-a)^{2}}{4} \Vert w \Vert _{[a,b],\infty} \mathcal{Q}^{1-\frac {1}{q}} \biggl\{ \biggl[\frac{1}{1+s} \bigl\vert f'(a) \bigr\vert ^{q}+ \biggl(2^{1-s}- \frac {1}{1+s} \biggr) \biggl\vert f' \biggl( \frac{a+b}{2} \biggr) \biggr\vert ^{q} \biggr]^{\frac{1}{q}} \\ &\qquad {} + \biggl[\frac{1}{1+s} \biggl\vert f' \biggl( \frac{a+b}{2} \biggr) \biggr\vert ^{q}+ \biggl(2^{1-s}- \frac{1}{1+s} \biggr) \bigl\vert f'(b) \bigr\vert ^{q} \biggr]^{\frac{1}{q}} \biggr\} . \end{aligned} $$

### Corollary 2.4

*Consider Theorem* 2.3.

(i) *If we take* $h(t)=1$, *then the following inequality for* $(m,P)$-*convex functions holds*:
  $$ \begin{aligned} & \biggl\vert \frac{1}{8(b-a)} \biggl[f(a)+6f \biggl(\frac{a+b}{2} \biggr)+f(b) \biggr] \int _{a}^{b}w(x)\,\mathrm{d}x-\frac{1}{b-a} \int_{a}^{b}w(x)f(x)\,\mathrm{d}x \biggr\vert \\ &\quad \leq\frac{b-a}{4} \Vert w \Vert _{[a,b],\infty} \mathcal{Q}^{1-\frac{1}{q}} \biggl\{ \biggl[ \bigl\vert f'(a) \bigr\vert ^{q}+m \biggl\vert f' \biggl(\frac{a+b}{2m} \biggr) \biggr\vert ^{q} \biggr]^{\frac{1}{q}} \\ &\qquad {} + \biggl[ \biggl\vert f' \biggl(\frac{a+b}{2} \biggr) \biggr\vert ^{q}+m \biggl\vert f' \biggl( \frac {b}{m} \biggr) \biggr\vert ^{q} \biggr]^{\frac{1}{q}} \biggr\} . \end{aligned} $$
(ii) *If we take* $h(t)=t(1-t)$ *and* $\alpha=1$, *then the following inequality for* $(m, tgs)$-*convex functions holds*:
  $$ \begin{aligned} & \biggl\vert \frac{1}{8(b-a)} \biggl[f(a)+6f \biggl(\frac{a+b}{2} \biggr)+f(b) \biggr] \int _{a}^{b}w(x)\,\mathrm{d}x-\frac{1}{b-a} \int_{a}^{b}w(x)f(x)\,\mathrm{d}x \biggr\vert \\ &\quad \leq\frac{b-a}{4} \Vert w \Vert _{[a,b],\infty} \mathcal{Q}^{1-\frac{1}{q}} \biggl(\frac{1}{6} \biggr)^{\frac{1}{q}} \biggl\{ \biggl[ \bigl\vert f'(a) \bigr\vert ^{q}+m \biggl\vert f' \biggl(\frac{a+b}{2m} \biggr) \biggr\vert ^{q} \biggr]^{\frac{1}{q}} \\ &\qquad {} + \biggl[ \biggl\vert f' \biggl(\frac{a+b}{2} \biggr) \biggr\vert ^{q}+m \biggl\vert f' \biggl( \frac {b}{m} \biggr) \biggr\vert ^{q} \biggr]^{\frac{1}{q}} \biggr\} . \end{aligned} $$
(iii) *If we take* $h(t)=\frac{\sqrt{1-t}}{2\sqrt{t}}$ *and* $\alpha=1$, *then the following inequality for* *m*-*MT*-*convex functions holds*:
  $$ \begin{aligned} & \biggl\vert \frac{1}{8(b-a)} \biggl[f(a)+6f \biggl(\frac{a+b}{2} \biggr)+f(b) \biggr] \int _{a}^{b}w(x)\,\mathrm{d}x-\frac{1}{b-a} \int_{a}^{b}w(x)f(x)\,\mathrm{d}x \biggr\vert \\ &\quad \leq\frac{b-a}{4} \Vert w \Vert _{[a,b],\infty} \mathcal{Q}^{1-\frac{1}{q}} \biggl(\frac{\pi}{4} \biggr)^{\frac{1}{q}} \biggl\{ \biggl[ \bigl\vert f'(a) \bigr\vert ^{q}+m \biggl\vert f' \biggl(\frac{a+b}{2m} \biggr) \biggr\vert ^{q} \biggr]^{\frac{1}{q}} \\ &\qquad {} + \biggl[ \biggl\vert f' \biggl(\frac{a+b}{2} \biggr) \biggr\vert ^{q}+m \biggl\vert f' \biggl( \frac {b}{m} \biggr) \biggr\vert ^{q} \biggr]^{\frac{1}{q}} \biggr\} . \end{aligned} $$

A similar result may be stated.

### Theorem 2.4

*Suppose that all assumptions of Theorem* 2.3 *are satisfied*. *Then*

2.13 $$ \begin{aligned}[b] & \biggl\vert \frac{1}{8(b-a)} \biggl[f(a)+6f \biggl(\frac{a+b}{2} \biggr)+f(b) \biggr] \int _{a}^{b}w(x)\,\mathrm{d}x-\frac{1}{b-a} \int_{a}^{b}w(x)f(x)\,\mathrm{d}x \biggr\vert \\ &\quad \leq\frac{b-a}{4} \Vert w \Vert _{[a,b],\infty} \mathcal{Q}^{1-\frac{1}{q}} \\ &\qquad {} \times \biggl\{ \biggl[ \int_{0}^{1} \biggl(h \biggl(1- \biggl( \frac{1-t}{2} \biggr)^{\alpha}\biggr)m \biggl\vert f' \biggl(\frac{a}{m} \biggr) \biggr\vert ^{q}+h \biggl( \biggl( \frac {1-t}{2} \biggr)^{\alpha}\biggr) \bigl\vert f'(b) \bigr\vert ^{q} \biggr)\,\mathrm{d}t \biggr]^{\frac{1}{q}} \\ &\qquad {} + \biggl[ \int_{0}^{1} \biggl(h \biggl( \biggl( \frac{t}{2} \biggr)^{\alpha}\biggr) \bigl\vert f'(a) \bigr\vert ^{q}+h \biggl(1- \biggl(\frac{t}{2} \biggr)^{\alpha}\biggr)m \biggl\vert f' \biggl( \frac{b}{m} \biggr) \biggr\vert ^{q} \biggr)\,\mathrm{d}t \biggr]^{\frac{1}{q}} \biggr\} . \end{aligned} $$

### Proof

The proof of Theorem 2.4 is analogous to that of Theorem 2.3 by using $ta+(1-t)\frac{a+b}{2}=\frac{1+t}{2}a+\frac{1-t}{2}b$ and $\frac{a+b}{2}t+(1-t)b=\frac{t}{2}a+(1-\frac{t}{2})b$. □

The following result holds for $(\alpha,m,s)$-convexity.

### Theorem 2.5

*Let* $f: \mathbb{R}_{0}\rightarrow\mathbb{R}$ *be a differentiable function on* $\mathbb{R}_{0}$, $a,b\in \mathbb{R}_{0}$, $a< b$, *let* $f'\in L^{1}[a,b]$, *and let* $w: [a,b]\rightarrow\mathbb{R}$ *be continuous and symmetric with respect to* $\frac{a+b}{2}$. *If* $|f'|^{q}$ *is* $(\alpha,m,s)$-*convex on* $[0,\frac{b}{m}]$ *for some fixed* $(\alpha ,m)\in(0,1]\times(0,1]$, $p^{-1}+q^{-1}=1$ *and* $q>1$, *then*

2.14 $$ \begin{aligned}[b] & \biggl\vert \frac{1}{8(b-a)} \biggl[f(a)+6f \biggl(\frac{a+b}{2} \biggr)+f(b) \biggr] \int _{a}^{b}w(x)\,\mathrm{d}x-\frac{1}{b-a} \int_{a}^{b}w(x)f(x)\,\mathrm{d}x \biggr\vert \\ &\quad \leq\frac{b-a}{4} \Vert w \Vert _{[a,b],\infty} \biggl( \frac {1+3^{p+1}}{(p+1)4^{p+1}} \biggr)^{\frac{1}{p}} \\ &\qquad {} \times \biggl\{ \biggl[\frac{1}{1+s\alpha} \bigl\vert f'(a) \bigr\vert ^{q}+ \biggl(2^{1-s}- \frac{1}{1+s\alpha} \biggr)m \biggl\vert f' \biggl( \frac{a+b}{2m} \biggr) \biggr\vert ^{q} \biggr]^{\frac{1}{q}} \\ &\qquad {} + \biggl[\frac{1}{1+s\alpha} \biggl\vert f' \biggl( \frac{a+b}{2} \biggr) \biggr\vert ^{q}+ \biggl(2^{1-s}- \frac{1}{1+s\alpha} \biggr)m \biggl\vert f' \biggl( \frac{b}{m} \biggr) \biggr\vert ^{q} \biggr]^{\frac{1}{q}} \biggr\} . \end{aligned} $$

### Proof

Since $|f'|^{q}$ is $(\alpha,m,s)$-convex on $[0,\frac{b}{m}]$, using the Hölder inequality for (2.3), we have

2.15 $$\begin{aligned}& \biggl\vert \frac{1}{8(b-a)} \biggl[f(a)+6f \biggl( \frac{a+b}{2} \biggr)+f(b) \biggr] \int_{a}^{b}w(x)\,\mathrm{d}x-\frac{1}{b-a} \int_{a}^{b}w(x)f(x)\,\mathrm{d}x \biggr\vert \\& \quad \leq\frac{b-a}{4} \Vert w \Vert _{[a,b],\infty} \biggl\{ \biggl( \int_{0}^{1} \biggl\vert \frac{3}{4}-t \biggr\vert ^{p}\,\mathrm{d}t \biggr)^{\frac{1}{p}} \biggl[ \int _{0}^{1} \biggl\vert f' \biggl(ta+(1-t)\frac{a+b}{2} \biggr) \biggr\vert ^{q}\, \mathrm{d}t \biggr]^{\frac{1}{q}} \\& \qquad {} + \biggl( \int_{0}^{1} \biggl\vert \frac{1}{4}-t \biggr\vert ^{p}\,\mathrm{d}t \biggr)^{\frac {1}{p}} \biggl[ \int_{0}^{1} \biggl\vert f' \biggl(t\frac{a+b}{2}+(1-t)b \biggr) \biggr\vert ^{q}\, \mathrm{d}t \biggr]^{\frac{1}{q}} \biggr\} \\& \quad \leq\frac{b-a}{4} \Vert w \Vert _{[a,b],\infty} \biggl( \frac {1+3^{p+1}}{(p+1)4^{p+1}} \biggr)^{\frac{1}{p}} \\& \qquad {} \times \biggl\{ \biggl[ \bigl\vert f'(a) \bigr\vert ^{q} \int_{0}^{1}t^{\alpha s}\,\mathrm{d}t+m \biggl\vert f' \biggl(\frac{a+b}{2m} \biggr) \biggr\vert ^{q} \int_{0}^{1}\bigl(1-t^{\alpha}\bigr)^{s}\,\mathrm{d}t \biggr]^{\frac{1}{q}} \\& \qquad {} + \biggl[ \biggl\vert f' \biggl(\frac{a+b}{2} \biggr) \biggr\vert ^{q} \int_{0}^{1}t^{\alpha s}\,\mathrm{d}t+m \biggl\vert f' \biggl(\frac{b}{m} \biggr) \biggr\vert ^{q} \int _{0}^{1}\bigl(1-t^{\alpha}\bigr)^{s}\,\mathrm{d}t \biggr]^{\frac{1}{q}} \biggr\} . \end{aligned}$$

From (2.15) we get the desired inequality in (2.14), since

$$ \begin{aligned} \int_{0}^{1}t^{\alpha s}\,\mathrm{d}t= \frac{1}{1+s\alpha}, \end{aligned} $$

and using the inequality $(1-t^{\alpha})^{s}\leq2^{1-s}-t^{s\alpha}$ for $t\in[0,1]$ with some fixed $\alpha\in(0,1]$, $s\in[0,1]$, we have

$$ \begin{aligned} \int_{0}^{1}\bigl(1-t^{\alpha}\bigr)^{s}\,\mathrm{d}t\leq \int_{0}^{1}\bigl(2^{1-s}-t^{\alpha s} \bigr)\,\mathrm{d}t=2^{1-s}-\frac{1}{1+s\alpha}. \end{aligned} $$

 □

Now, we point out a particular case of Theorem 2.5.

### Corollary 2.5

*If we take* $s=1$ *and* $m=1=\alpha$ *in Theorem* 2.5, *then the following inequality for convex functions holds*:

$$ \begin{aligned} & \biggl\vert \frac{1}{8(b-a)} \biggl[f(a)+6f \biggl(\frac{a+b}{2} \biggr)+f(b) \biggr] \int _{a}^{b}w(x)\,\mathrm{d}x-\frac{1}{b-a} \int_{a}^{b}w(x)f(x)\,\mathrm{d}x \biggr\vert \\ &\quad \leq\frac{b-a}{2^{2+\frac{1}{q}}} \Vert w \Vert _{[a,b],\infty} \biggl( \frac {1+3^{p+1}}{(p+1)4^{p+1}} \biggr)^{\frac{1}{p}} \biggl\{ \biggl[ \bigl\vert f'(a) \bigr\vert ^{q}+ \biggl\vert f' \biggl(\frac{a+b}{2} \biggr) \biggr\vert ^{q} \biggr]^{\frac{1}{q}} \\ &\qquad {} + \biggl[ \biggl\vert f' \biggl(\frac{a+b}{2} \biggr) \biggr\vert ^{q}+ \bigl\vert f'(b) \bigr\vert ^{q} \biggr]^{\frac{1}{q}} \biggr\} . \end{aligned} $$

Next, we would like to point out some published results that are particular cases of the obtained main results.

### Remark 2.4

In Lemma 2.1, if we take $w(x) =1$, then identity (2.1) becomes the following equation proved by Shuang et al. [28]:

$$ \begin{aligned} &\frac{1}{8} \biggl[f(a)+6f \biggl( \frac{a+b}{2} \biggr)+f(b) \biggr]-\frac {1}{b-a} \int_{a}^{b}f(x)\,\mathrm{d}x \\ &\quad =\frac{b-a}{4} \int_{0}^{1} \biggl[ \biggl(\frac{3}{4}-t \biggr)f' \biggl(ta+(1-t)\frac{a+b}{2} \biggr)+ \biggl( \frac{1}{4}-t \biggr)f' \biggl(t\frac {a+b}{2}+(1-t)b \biggr) \biggr]\,\mathrm{d}t.\end{aligned} $$

### Remark 2.5

If we take $h(t)=t$ and $w(x)=1$ in Theorems 2.1 and 2.3, then we obtain Theorems 3.1 and 3.5 established by Shuang et al. [28], respectively.

## Further estimation results

If the considered function $f'$ is bounded from below and above, then we have the following result.

### Theorem 3.1

*Let* $f: I\subseteq\mathbb{R}\rightarrow\mathbb{R}$ *be a differentiable function on* $I^{\circ}$, $a,b\in I^{\circ}$, $a< b$, *and let* $w: [a,b]\rightarrow\mathbb{R}$ *be continuous and symmetric with respect to* $\frac{a+b}{2}$. *Assume that* $f'$ *is integrable on* $[a,b]$ *and there exist constants* $m < M$ *such that* $-\infty< m\leq f'(x)\leq M<+\infty$ *for all* $x\in[a,b]$. *Then*

3.1 $$ \begin{aligned}[b] & \biggl\vert \frac{1}{8(b-a)} \biggl[f(a)+6f \biggl(\frac{a+b}{2} \biggr)+f(b) \biggr] \int _{a}^{b}w(x)\,\mathrm{d}x-\frac{1}{b-a} \int_{a}^{b}w(x)f(x)\,\mathrm{d}x \\ &\qquad {} -\frac{(b-a)(m+M)}{8} \biggl[ \int_{0}^{1}p_{1}(t)\,\mathrm{d}t+ \int _{0}^{1}p_{2}(t)\,\mathrm{d}t \biggr] \biggr\vert \\ &\quad \leq\frac{5(b-a)(M-m)}{64} \Vert w \Vert _{[a,b],\infty}, \end{aligned} $$

*where* $p_{1}(t)$ *and* $p_{2}(t)$ *are defined in Lemma* 2.1.

### Proof

From Lemma 2.1 we have

$$ \begin{aligned} &\frac{1}{8(b-a)} \biggl[f(a)+6f \biggl( \frac{a+b}{2} \biggr)+f(b) \biggr] \int _{a}^{b}w(x)\,\mathrm{d}x-\frac{1}{b-a} \int_{a}^{b}w(x)f(x)\,\mathrm{d}x \\ &\quad =\frac{b-a}{4} \biggl\{ \int_{0}^{1}p_{1}(t) \biggl[f' \biggl(ta+(1-t)\frac {a+b}{2} \biggr)- \frac{m+M}{2}+\frac{m+M}{2} \biggr]\,\mathrm{d}t \\ &\qquad {} + \int_{0}^{1}p_{2}(t) \biggl[f' \biggl(t\frac{a+b}{2}+(1-t)b \biggr)- \frac {m+M}{2}+\frac{m+M}{2} \biggr]\,\mathrm{d}t \biggr\} \\ &\quad =\frac{b-a}{4} \biggl\{ \int_{0}^{1}p_{1}(t) \biggl[f' \biggl(ta+(1-t)\frac {a+b}{2} \biggr)- \frac{m+M}{2} \biggr]\,\mathrm{d}t \\ &\qquad {} + \int_{0}^{1}p_{2}(t) \biggl[f' \biggl(t\frac{a+b}{2}+(1-t)b \biggr)- \frac {m+M}{2} \biggr]\,\mathrm{d}t \biggr\} \\ &\qquad {} +\frac{(b-a)(m+M)}{8} \biggl\{ \int_{0}^{1}p_{1}(t)\,\mathrm{d}t + \int_{0}^{1}p_{2}(t)\,\mathrm{d}t \biggr\} . \end{aligned} $$

So

$$ \begin{aligned} \mathcal{T}:={}&\frac{1}{8(b-a)} \biggl[f(a)+6f \biggl(\frac{a+b}{2} \biggr)+f(b) \biggr] \int_{a}^{b}w(x)\,\mathrm{d}x-\frac{1}{b-a} \int _{a}^{b}w(x)f(x)\,\mathrm{d}x \\ & {} -\frac{(b-a)(m+M)}{8} \biggl[ \int_{0}^{1}p_{1}(t)\,\mathrm{d}t+ \int _{0}^{1}p_{2}(t)\,\mathrm{d}t \biggr] \\ ={}&\frac{b-a}{4} \biggl\{ \int_{0}^{1}p_{1}(t) \biggl[f' \biggl(ta+(1-t)\frac {a+b}{2} \biggr)- \frac{m+M}{2} \biggr]\,\mathrm{d}t \\ & {} + \int_{0}^{1}p_{2}(t) \biggl[f' \biggl(t\frac{a+b}{2}+(1-t)b \biggr)- \frac {m+M}{2} \biggr]\,\mathrm{d}t \biggr\} . \end{aligned} $$

Therefore

$$ \begin{aligned} \vert \mathcal{T} \vert \leq{}&\frac{b-a}{4} \biggl\{ \int_{0}^{1} \bigl\vert p_{1}(t) \bigr\vert \biggl\vert f' \biggl(ta+(1-t)\frac{a+b}{2} \biggr)- \frac{m+M}{2} \biggr\vert \,\mathrm{d}t \\ & {}+ \int_{0}^{1} \bigl\vert p_{2}(t) \bigr\vert \biggl\vert f' \biggl(t\frac{a+b}{2}+(1-t)b \biggr)- \frac{m+M}{2} \biggr\vert \,\mathrm{d}t \biggr\} \\ \leq{}&\frac{(b-a)(M-m)}{8} \biggl\{ \int_{0}^{1} \bigl\vert p_{1}(t) \bigr\vert \,\mathrm{d}t+ \int_{0}^{1} \bigl\vert p_{2}(t) \bigr\vert \,\mathrm{d}t \biggr\} . \end{aligned} $$

Since $f'$ satisfies $-\infty< m\leq f'(x)\leq M<+\infty$, we have

$$ \begin{aligned} m-\frac{m+M}{2}\leq f'(x)- \frac{m+M}{2}\leq M-\frac{m+M}{2}, \end{aligned} $$

which implies that

$$ \begin{aligned} \biggl\vert f'(x)-\frac{m+M}{2} \biggr\vert \leq\frac{M-m}{2}. \end{aligned} $$

Also, since *w* is symmetric with respect to $\frac{a+b}{2}$, we get

$$ \begin{aligned} \vert \mathcal{T} \vert \leq&\frac{(b-a)(M-m)}{8} \\ & {}\times \biggl\{ \int_{0}^{1} \biggl\vert \frac{3}{4} \int_{0}^{1}w \biggl(sa+(1-s)\frac {a+b}{2} \biggr)\,\mathrm{d}s- \int_{0}^{t}w \biggl(sa+(1-s)\frac{a+b}{2} \biggr)\,\mathrm{d}s \biggr\vert \,\mathrm{d}t \\ & {} + \int_{0}^{1} \biggl\vert \frac{1}{4} \int_{0}^{1}w \biggl(s\frac{a+b}{2}+(1-s)b \biggr)\,\mathrm{d}s- \int_{0}^{t}w \biggl(s\frac{a+b}{2}+(1-s)b \biggr)\,\mathrm{d}s \biggr\vert \,\mathrm{d}t \biggr\} \\ \leq{}&\frac{(b-a)(M-m)}{8} \Vert w \Vert _{[a,b],\infty} \\ & {} \times \biggl\{ \int_{0}^{1} \biggl\vert \frac{3}{4} \int_{0}^{1}\,\mathrm{d}s- \int _{0}^{t}\,\mathrm{d}s \biggr\vert \, \mathrm{d}t+ \int_{0}^{1} \biggl\vert \frac{1}{4} \int _{0}^{1}\,\mathrm{d}s- \int_{0}^{t}\,\mathrm{d}s \biggr\vert \, \mathrm{d}t \biggr\} \\ \leq{}&\frac{5(b-a)(M-m)}{64} \Vert w \Vert _{[a,b],\infty}. \end{aligned} $$

This ends the proof. □

### Corollary 3.1

*In Theorem* 3.2, *if* $w(x)=1$, *then we get*

3.2 $$ \begin{aligned} \biggl\vert \frac{1}{8} \biggl[f(a)+6f \biggl(\frac{a+b}{2} \biggr)+f(b) \biggr]-\frac {1}{b-a} \int_{a}^{b}f(x)\,\mathrm{d}x \biggr\vert &\leq \frac{(b-a)(9M-m)}{64}. \end{aligned} $$

### Proof

If we take $w(x)=1$, then the relation $\|w\| _{[a,b],\infty}=1$ implies that

$$ \begin{aligned} & \biggl\vert \frac{1}{8} \biggl[f(a)+6f \biggl(\frac{a+b}{2} \biggr)+f(b) \biggr]-\frac {1}{b-a} \int_{a}^{b}f(x)\,\mathrm{d}x \biggr\vert \\ &\quad \leq\frac{(b-a)(m+M)}{8} \biggl\vert \int_{0}^{1}p_{1}(t)\,\mathrm{d}t+ \int _{0}^{1}p_{2}(t)\,\mathrm{d}t \biggr\vert +\frac{5(b-a)(M-m)}{64} \\ &\quad \leq\frac{(b-a)(m+M)}{8} \biggl[ \biggl\vert \int_{0}^{1}p_{1}(t)\,\mathrm{d}t \biggr\vert + \biggl\vert \int_{0}^{1}p_{2}(t)\,\mathrm{d}t \biggr\vert \biggr]+\frac{5(b-a)(M-m)}{64} \\ &\quad \leq\frac{(b-a)(m+M)}{16}+\frac{5(b-a)(M-m)}{64} \\ &\quad =\frac{(b-a)(9M-m)}{64}. \end{aligned} $$

 □

Our next goal is an estimation-type result with respect to the weighted Simpson-like type inequality when the derivative of the considered function $f'$ satisfies a Lipschitz condition.

### Theorem 3.2

*Let* $f: I\subseteq\mathbb{R}\rightarrow\mathbb{R}$ *be a differentiable function on* $I^{\circ}$, $a,b\in I^{\circ}$, $a< b$, *and let* $w: [a,b]\rightarrow\mathbb{R}$ *be continuous and symmetric with respect to* $\frac{a+b}{2}$. *Assume that* $f'$ *is integrable on* $[a,b]$ *and satisfies a Lipschitz condition for some* $L>0$. *Then*

3.3 $$ \begin{aligned}[b] & \biggl\vert \frac{1}{8(b-a)} \biggl[f(a)+6f \biggl(\frac{a+b}{2} \biggr)+f(b) \biggr] \int _{a}^{b}w(x)\,\mathrm{d}x-\frac{1}{b-a} \int_{a}^{b}w(x)f(x)\,\mathrm{d}x \\ &\qquad {} -\frac{b-a}{4} \biggl[f'(a) \int_{0}^{1}p_{1}(t)\, \mathrm{d}t+f'(b) \int _{0}^{1}p_{2}(t)\,\mathrm{d}t \biggr] \biggr\vert \\ &\quad \leq\frac{41(b-a)^{2}L}{3\cdot2^{8}} \Vert w \Vert _{[a,b],\infty}, \end{aligned} $$

*where* $p_{1}(t)$ *and* $p_{2}(t)$ *are defined in Lemma* 2.1.

### Proof

From Lemma 2.1 we have

$$ \begin{aligned} &\frac{1}{8(b-a)} \biggl[f(a)+6f \biggl( \frac{a+b}{2} \biggr)+f(b) \biggr] \int _{a}^{b}w(x)\,\mathrm{d}x-\frac{1}{b-a} \int_{a}^{b}w(x)f(x)\,\mathrm{d}x \\ &\quad =\frac{b-a}{4} \biggl\{ \int_{0}^{1}p_{1}(t) \biggl[f' \biggl(ta+(1-t)\frac {a+b}{2} \biggr)-f'(a)+f'(a) \biggr]\,\mathrm{d}t \\ &\qquad {} + \int_{0}^{1}p_{2}(t) \biggl[f' \biggl(t\frac{a+b}{2}+(1-t)b \biggr)-f'(b)+f'(b) \biggr]\,\mathrm{d}t \biggr\} \\ &\quad =\frac{b-a}{4} \biggl\{ \int_{0}^{1}p_{1}(t) \biggl[f' \biggl(ta+(1-t)\frac {a+b}{2} \biggr)-f'(a) \biggr]\,\mathrm{d}t \\ &\qquad {} + \int_{0}^{1}p_{2}(t) \biggl[f' \biggl(t\frac{a+b}{2}+(1-t)b \biggr)-f'(b) \biggr]\,\mathrm{d}t \biggr\} \\ &\qquad {} +\frac{b-a}{4} \biggl\{ \int_{0}^{1}p_{1}(t)f'(a)\, \mathrm{d}t + \int_{0}^{1}p_{2}(t)f'(b)\, \mathrm{d}t \biggr\} . \end{aligned} $$

Then

$$ \begin{aligned} \mathcal{R}={}&\frac{1}{8(b-a)} \biggl[f(a)+6f \biggl( \frac{a+b}{2} \biggr)+f(b) \biggr] \int_{a}^{b}w(x)\,\mathrm{d}x-\frac{1}{b-a} \int _{a}^{b}w(x)f(x)\,\mathrm{d}x \\ &{} -\frac{b-a}{4} \biggl[ \int_{0}^{1}p_{1}(t)f'(a)\, \mathrm{d}t + \int_{0}^{1}p_{2}(t)f'(b)\, \mathrm{d}t \biggr] \\ ={}&\frac{b-a}{4} \biggl\{ \int_{0}^{1}p_{1}(t) \biggl[f' \biggl(ta+(1-t)\frac {a+b}{2} \biggr)-f'(a) \biggr]\,\mathrm{d}t \\ &{} + \int_{0}^{1}p_{2}(t) \biggl[f' \biggl(t\frac{a+b}{2}+(1-t)b \biggr)-f'(b) \biggr]\,\mathrm{d}t \biggr\} . \end{aligned} $$

Since $f'$ satisfies Lipschitz conditions for some $L> 0$, we have

$$ \begin{aligned} \biggl\vert f' \biggl(ta+(1-t) \frac{a+b}{2} \biggr)-f'(a) \biggr\vert \leq L \biggl\vert ta+(1-t)\frac{a+b}{2}-a \biggr\vert =L \vert 1-t \vert \biggl( \frac{b-a}{2} \biggr) \end{aligned} $$

and

$$ \begin{aligned} \biggl\vert f' \biggl(t \frac{a+b}{2}+(1-t)b \biggr)-f'(b) \biggr\vert \leq L \biggl\vert t\frac{a+b}{2}+(1-t)b-b \biggr\vert =L \vert t \vert \biggl( \frac{b-a}{2} \biggr). \end{aligned} $$

Hence

$$ \begin{aligned} \vert \mathcal{R} \vert \leq\frac{(b-a)^{2}L}{8} \biggl\{ \int_{0}^{1}(1-t) \bigl\vert p_{1}(t) \bigr\vert \,\mathrm{d}t+ \int_{0}^{1}t \bigl\vert p_{2}(t) \bigr\vert \,\mathrm{d}t \biggr\} . \end{aligned} $$

Also, since *w* is symmetric with respect to $\frac{a+b}{2}$, we get

$$ \begin{aligned} \vert \mathcal{R} \vert &\leq\frac{(b-a)^{2}L}{8} \Vert w \Vert _{[a,b],\infty} \biggl\{ \int_{0}^{1}(1-t) \biggl\vert \frac{3}{4}-t \biggr\vert \,\mathrm{d}t+ \int_{0}^{1}t \biggl\vert \frac {1}{4}-t \biggr\vert \,\mathrm{d}t \biggr\} \\ &\leq\frac{41(b-a)^{2}L}{3\cdot2^{8}} \Vert w \Vert _{[a,b],\infty}. \end{aligned} $$

This completes the proof. □

### Corollary 3.2

*In Theorem* 3.2, *if* $w(x)=1$, *then we get*

3.4 $$ \begin{aligned} [b]& \biggl\vert \frac{1}{8} \biggl[f(a)+6f \biggl(\frac{a+b}{2} \biggr)+f(b) \biggr]-\frac {1}{b-a} \int_{a}^{b}f(x)\,\mathrm{d}x \biggr\vert \\ &\quad \leq\frac{41(b-a)^{2}L}{3\cdot2^{8}}+\frac{b-a}{16} \bigl[f'(a)+f'(b) \bigr]. \end{aligned} $$

## Applications

### *f*-divergence measures

In various applications of probability theory, one of the primary themes is discovering a proper measure of distance between any two probability distributions. Let a set *ψ* and a *σ*-finite measure *μ* be given and consider the set of all probability densities on *μ* defined on

$$\varOmega:= \biggl\{ \rho|\rho: \psi\rightarrow\mathbb{R}, \rho(x) > 0, \int_{\psi}\rho(x)\,\mathrm{d}\mu(x) = 1 \biggr\} . $$

Let $f:(0,\infty) \rightarrow\mathbb{R} $ be a given mapping and consider $D_{f}(\rho,\tau) $ defined by

4.1 $$ \begin{aligned} D_{f}(\rho,\tau):= \int_{\psi}\rho(x)f \biggl[\frac{\tau(x)}{\rho(x)} \biggr]\,\mathrm{d} \mu(x), \quad \rho,\tau\in\varOmega. \end{aligned} $$

If *f* is convex, then (4.1) is known as the Csisźar *f*-divergence [4].

Shioya and Da-te [27] presented the Hermite–Hadamard $(HH)$ divergence

4.2 $$ \begin{aligned} D^{f}_{HH}(\rho, \tau)= \int_{\psi}\rho(x)\frac{\int^{\frac{\tau(x)}{\rho (x)}}_{1}f(t)\,\mathrm{d}t}{\frac{\tau(x)}{\rho(x)}-1}\,\mathrm{d}\mu(x),\quad \rho , \tau\in\varOmega, \end{aligned} $$

where *f* is convex on $(0,\infty)$ with $f(1) = 0$. In the same paper [27], they also gave the property of *HH* divergence that $D^{f}_{HH}(\rho; \tau) \geq0$ with equality if and only if $\rho= \tau$.

#### Proposition 4.1

*Let all assumptions of Theorem* 2.5 *hold with* $f(1)=0 $. *If* *ρ*, $\tau\in\varOmega$, *then*

4.3 $$ \begin{aligned}[b] & \biggl\vert \frac{1}{8} \biggl[D_{f}(\rho,\tau)+6 \int_{\psi}\rho(x)f \biggl(\frac {\rho(x)+\tau(x)}{2\rho(x)} \biggr)\,\mathrm{d} \mu(x) \biggr]-D^{f}_{HH}(\rho ,\tau) \biggr\vert \\ &\quad \leq \biggl(\frac{7}{3\cdot2^{9}} \biggr)^{\frac{1}{2}} \biggl\{ \biggl[ \bigl\vert f'(1) \bigr\vert ^{2} \int_{\psi}\frac{ (\tau(x)-\rho(x) )^{2}}{\rho (x)}\,\mathrm{d}\mu(x) \\ &\qquad {} + \int_{\psi}\frac{ (\tau(x)-\rho(x) )^{2}}{\rho(x)} \biggl\vert f' \biggl(\frac{\rho(x)+\tau(x)}{2\rho(x)} \biggr) \biggr\vert ^{2}\,\mathrm{d}\mu(x) \biggr]^{\frac{1}{2}} \\ &\qquad {} + \biggl[ \int_{\psi}\frac{ (\tau(x)-\rho(x) )^{2}}{\rho(x)} \biggl\vert f' \biggl(\frac{\rho(x)+\tau(x)}{2\rho(x)} \biggr) \biggr\vert ^{2}\,\mathrm{d}\mu (x) \\ &\qquad {} + \int_{\psi}\frac{ (\tau(x)-\rho(x) )^{2}}{\rho(x)} \biggl\vert f' \biggl(\frac{\tau(x)}{\rho(x)} \biggr) \biggr\vert ^{2}\,\mathrm{d}\mu(x) \biggr]^{\frac {1}{2}} \biggr\} . \end{aligned} $$

#### Proof

Let $\varPsi_{1} = \{x\in\psi:\tau(x)>\rho(x) \}$, $\varPsi_{2} = \{x\in\psi:\tau(x)<\rho(x) \}$, and $\varPsi_{3} = \{x\in\psi:\tau(x)=\rho(x) \}$.

Obviously, if $x\in\varPsi_{3}$, then equality holds in (4.3). Now if we take $w(x)=1$ and $q=2$ in Corollary 2.5, then for $a=1$, $b=\frac{\tau(x)}{\rho(x)}$, and $x\in \varPsi_{1}$, multiplying both sides of the obtained results by $\rho(x)$ and then integrating on $\varPsi_{1}$, we get

4.4 $$ \begin{aligned}[b] & \biggl\vert \frac{1}{8} \biggl[ \int_{\varPsi_{1}} \rho(x)f \biggl(\frac{\tau (x)}{\rho(x)} \biggr)\, \mathrm{d}\mu(x)+6 \int_{\varPsi_{1}} \rho(x)f \biggl(\frac{ \rho(x)+\tau(x)}{2 \rho(x)} \biggr)\, \mathrm{d}\mu(x) \biggr] \\ &\qquad {} - \int_{\varPsi_{1}} \rho(x)\frac{\int^{\frac{\tau(x)}{ \rho (x)}}_{1}f(t)\,\mathrm{d}t}{\frac{\tau(x)}{ \rho(x)}-1}\,\mathrm{d}\mu(x) \biggr\vert \\ &\quad \leq \biggl(\frac{7}{3\cdot2^{9}} \biggr)^{\frac{1}{2}} \biggl\{ \biggl[ \bigl\vert f'(1) \bigr\vert ^{2} \int_{\varPsi_{1}}\frac{ (\tau(x)- \rho(x) )^{2}}{ \rho(x)}\,\mathrm{d}\mu(x) \\ &\qquad {} + \int_{\varPsi_{1}}\frac{ (\tau(x)- \rho(x) )^{2}}{ \rho(x)} \biggl\vert f' \biggl(\frac{ \rho(x)+\tau(x)}{2 \rho(x)} \biggr) \biggr\vert ^{2}\,\mathrm{d}\mu (x) \biggr]^{\frac{1}{2}} \\ &\qquad {} + \biggl[ \int_{\varPsi_{1}}\frac{ (\tau(x)- \rho(x) )^{2}}{ \rho (x)} \biggl\vert f' \biggl(\frac{ \rho(x)+\tau(x)}{2 \rho(x)} \biggr) \biggr\vert ^{2}\,\mathrm{d}\mu(x) \\ & \qquad {}+ \int_{\varPsi_{1}}\frac{ (\tau(x)- \rho(x) )^{2}}{ \rho(x)} \biggl\vert f' \biggl(\frac{\tau(x)}{ \rho(x)} \biggr) \biggr\vert ^{2}\,\mathrm{d}\mu(x) \biggr]^{\frac{1}{2}} \biggr\} .\end{aligned} $$

Similarly, if $x\in\varPsi_{2}$, then using Corollary 2.5 for $a=\frac{\tau(x)}{\rho(x)}$, $b=1$, multiplying both sides of the obtained results by $\rho(x)$, and integrating on $\varPsi_{2}$, we get

4.5 $$\begin{aligned}& \biggl\vert \frac{1}{8} \biggl[ \int_{\varPsi_{2}} \rho(x)f \biggl(\frac{\tau (x)}{ \rho(x)} \biggr)\, \mathrm{d}\mu(x)+6 \int_{\varPsi_{2}} \rho(x)f \biggl(\frac{ \rho(x)+\tau(x)}{2 \rho(x)} \biggr)\, \mathrm{d}\mu(x) \biggr] \\& \qquad {} - \int_{\varPsi_{2}} \rho(x)\frac{\int^{\frac{\tau(x)}{ \rho (x)}}_{1}f(t)\,\mathrm{d}t}{\frac{\tau(x)}{ \rho(x)}-1}\,\mathrm{d}\mu(x) \biggr\vert \\& \quad \leq \biggl(\frac{7}{3\cdot2^{9}} \biggr)^{\frac{1}{2}} \biggl\{ \biggl[ \bigl\vert f'(1) \bigr\vert ^{2} \int_{\varPsi_{2}}\frac{ (\tau(x)- \rho(x) )^{2}}{ \rho(x)}\,\mathrm{d}\mu(x) \\& \qquad {}+ \int_{\varPsi_{2}}\frac{ (\tau(x)- \rho(x) )^{2}}{ \rho(x)} \biggl\vert f' \biggl(\frac{ \rho(x)+\tau(x)}{2 \rho(x)} \biggr) \biggr\vert ^{2}\,\mathrm{d}\mu (x) \biggr]^{\frac{1}{2}} \\& \qquad {} + \biggl[ \int_{\varPsi_{2}}\frac{ (\tau(x)- \rho(x) )^{2}}{ \rho (x)} \biggl\vert f' \biggl(\frac{ \rho(x)+\tau(x)}{2 \rho(x)} \biggr) \biggr\vert ^{2}\,\mathrm{d}\mu(x) \\& \qquad {} + \int_{\varPsi_{2}}\frac{ (\tau(x)- \rho(x) )^{2}}{ \rho(x)} \biggl\vert f' \biggl(\frac{\tau(x)}{ \rho(x)} \biggr) \biggr\vert ^{2}\,\mathrm{d}\mu(x) \biggr]^{\frac{1}{2}} \biggr\} . \end{aligned}$$

Adding inequalities (4.4) and (4.5) and then using the triangle inequality, we get the desired result. □

### Random variable

Suppose that for $0 < a < b$, $w: [a, b]\rightarrow[0,+\infty)$ is a continuous probability density of a continuous random variable *X* that is symmetric about $\frac{a+b}{2}$. Also, for $r\in\mathbb{R}$, suppose that the *r*th moment

4.6 $$ E_{r}(X)= \int^{b}_{a}x^{r} w(x)\,\mathrm{d}x $$

is finite.

Since *w* is symmetric and $\int^{b}_{a}w(x)\,\mathrm{d}x=1$, we have

4.7 $$ E(X)= \int^{b}_{a}xw(x)\,\mathrm{d}x=\frac{a+b}{2}, $$

which follows from

$$\begin{aligned} \int^{b}_{a}xw(x)\,\mathrm{d}x =& \int^{b}_{a}(b+a-x)w(b+a-x)\,\mathrm{d}x\\ =& \int ^{b}_{a}(b+a-x)w(x)\,\mathrm{d}x. \end{aligned}$$

Based on the above-mentioned derivations, we obtain the following estimates of the *r*th moment.

- If we consider $f(x)=x^{r}$ on $[a,b]$ for $r\geq2$, then the function $|f'(x) |^{q}=r^{q}x^{q(r-1)}$ with $q>1$ is a convex function. Therefore, using this function in Remark 2.3 with $s=1$ and in Corollary 2.5, respectively, we have
  $$ \begin{aligned} & \bigl\vert \bigl(E(X) \bigr)^{r}-E_{r}(X) \bigr\vert \\ &\quad \leq\frac{r(b-a)^{2}}{2^{2+\frac{1}{q}}} \Vert w \Vert _{[a,b],\infty}\mathcal {Q}^{1-\frac{1}{q}} \\ & \qquad {}\times \biggl\{ \biggl[a^{q(r-1)}+ \biggl(\frac{a+b}{2} \biggr)^{q(r-1)} \biggr]^{\frac{1}{q}} + \biggl[ \biggl( \frac{a+b}{2} \biggr)^{q(r-1)}+b^{q(r-1)} \biggr]^{\frac{1}{q}} \biggr\} \end{aligned} $$
  and
  $$\begin{aligned}& \biggl\vert \frac{a^{r}+b^{r}}{8}+\frac{3}{4} \bigl(E(X) \bigr)^{r}-E_{r}(X) \biggr\vert \\& \quad \leq\frac{r(b-a)^{2}}{2^{2+\frac{1}{q}}} \Vert w \Vert _{[a,b],\infty} \biggl( \frac {1+3^{p+1}}{(p+1)4^{p+1}} \biggr)^{\frac{1}{p}} \\& \qquad {}\times \biggl\{ \biggl[a^{q(r-1)}+ \biggl(\frac{a+b}{2} \biggr)^{q(r-1)} \biggr]^{\frac{1}{q}} + \biggl[ \biggl( \frac{a+b}{2} \biggr)^{q(r-1)}+b^{q(r-1)} \biggr]^{\frac{1}{q}} \biggr\} . \end{aligned}$$
- If we consider $f(x)=x^{r}$ on $[a,b]$ for $r\in\mathbb{R}$, then $m=ra^{r-1}\leq f'(x)=rx^{r-1}\leq rb^{r-1}=M$, and so from (3.2) in Corollary 3.1 we have
  $$ \begin{aligned} & \biggl\vert \frac{a^{r}+b^{r}}{8}+\frac{3}{4} \bigl(E(X) \bigr)^{r}-E_{r}(X) \biggr\vert \leq \frac{r(9b^{r-1}-a^{r-1})(b-a)}{64}. \end{aligned} $$
- If we consider $f(x)=x^{r}$ on $[a,b]$ for $r\in\mathbb{R}$, then the Lipschitz constant $L=\sup_{x\in[a,b]}|f'(x)|=\sup_{x\in[a,b]}rx^{r-1}$ is equivalent to
  $$ L=\textstyle\begin{cases} rb^{r-1}, &r\geq1,\\ ra^{r-1}, & r< 1. \end{cases}$$

So from (3.4) in Corollary 3.2 we have

$$ \begin{aligned} \biggl\vert \frac{a^{r}+b^{r}}{8}+\frac{3}{4} \bigl(E(X) \bigr)^{r}-E_{r}(X) \biggr\vert = \textstyle\begin{cases} \frac{r(b-a)}{16} [a^{r-1}+ (1+\frac{41(b-a)}{48} )b^{r-1} ], & r\geq1, \\ \frac{r(b-a)}{16} [ (1+\frac{41(b-a)}{48} )a^{r-1}+b^{r-1} ], & r< 1. \end{cases}\displaystyle \end{aligned} $$

#### Remark 4.1

Applications based on the obtained results to special means can be given, and we omit the details.

## Conclusions

Based on a new weighted Simpson-like type integral identity, we obtained certain estimation-type results with respect to the weighted Simpson-like type inequality for the first-order differentiable mappings. Some particular cases are considered, which can be derived from the main results in the present paper. It is an interesting topic to apply these estimations to *f*-divergence measures and to higher moments of continuous random variables.
